# Stimulation of Toll-Like Receptor 3 Diminishes Intracellular Growth of *Salmonella* Typhimurium by Enhancing Autophagy in Murine Macrophages

**DOI:** 10.3390/metabo11090602

**Published:** 2021-09-04

**Authors:** Hyo-Ji Lee, Sun-Hye Lee, Ji-Hui Jeon, Hyo-Jung Kim, Eui-Kwon Jeong, Min-Jeong Kim, Young Mee Jung, Yu-Jin Jung

**Affiliations:** 1 Department of Biological Sciences, Kangwon National University, Chuncheon 24341, Korea; atonaema@kangwon.ac.kr; 2 Kangwon Radiation Convergence Research Support Center, Kangwon National University, Chuncheon 24341, Korea; ymjung@kangwon.ac.kr; 3 BIT Medical Convergence Graduate Program, Kangwon National University, Chuncheon 24341, Korea; lsh0805@naver.com (S.-H.L.); 202016224@kangwon.ac.kr (H.-J.K.); jek7539@kangwon.ac.kr (E.-K.J.); jjeong1012@kangwon.ac.kr (M.-J.K.); 4 Department of Chemistry, Kangwon National University, Chuncheon 24341, Korea

**Keywords:** autophagy, macrophage, reactive oxygen species (ROS), *Salmonella enterica* serovar Typhimurium, Toll-like receptor 3

## Abstract

The *Salmonella enterica* serovar Typhimurium (*S.* Typhimurium) is a facultative Gram-negative bacterium that causes acute gastroenteritis and food poisoning. *S*. Typhimurium can survive within macrophages that are able to initiate the innate immune response after recognizing bacteria via various pattern-recognition receptors (PRRs), such as Toll-like receptors (TLRs). In this study, we investigated the effects and molecular mechanisms by which agonists of endosomal TLRs—especially TLR3—contribute to controlling *S*. Typhimurium infection in murine macrophages. Treatment with polyinosinic:polycytidylic acid (poly(I:C))—an agonist of TLR3—significantly suppressed intracellular bacterial growth by promoting intracellular ROS production in *S*. Typhimurium-infected cells. Pretreatment with diphenyleneiodonium (DPI)—an NADPH oxidase inhibitor—reduced phosphorylated MEK1/2 levels and restored intracellular bacterial growth in poly(I:C)-treated cells during *S*. Typhimurium infection. Nitric oxide (NO) production increased through the NF-κB-mediated signaling pathway in poly(I:C)-treated cells during *S*. Typhimurium infection. Intracellular microtubule-associated protein 1A/1B-light chain 3 (LC3) levels were increased in poly(I:C)-treated cells; however, they were decreased in cells pretreated with 3-methyladenine (3-MA)—a commonly used inhibitor of autophagy. These results suggest that poly(I:C) induces autophagy and enhances ROS production via MEK1/2-mediated signaling to suppress intracellular bacterial growth in *S*. Typhimurium-infected murine macrophages, and that a TLR3 agonist could be developed as an immune enhancer to protect against *S*. Typhimurium infection.

## 1. Introduction

The *Salmonella enterica* serovar Typhimurium (*S.* Typhimurium) is a Gram-negative pathogen that is associated with gastroenteritis and severe systemic disease in humans, resembling typhoid fever in mice [1]. *S*. Typhimurium ingested through contaminated food or water can migrate from the lumen to the lamina propria via various processes, including transcytosis through microfold (M) cells, uptake by CXCR-positive macrophages/dendritic cells (DCs), and *Salmonella* pathogenicity island 1 (SPI-1)-mediated uptake [2,3]. In the intestinal lamina propria, *S*. Typhimurium can be recognized and internalized by various phagocytic cells, including macrophages and DCs [4,5]. These cells remove *S*. Typhimurium via phagocytosis and recruit other immune cells to the infection site by secreting proinflammatory cytokines and chemokines. During *S*. Typhimurium infection, innate immune receptors—including Toll-like receptors (TLRs), NOD-like receptors (NLRs), and RIG-I-like receptors (RLRs)—recognize conserved microbial ligands called pathogen-associated molecular patterns (PAMPs) [6]. *S*. Typhimurium expresses various ligands for TLRs—such as lipopolysaccharide (LPS), flagellin, double-stranded RNA (dsRNA), and CpG DNA—and these ligands lead to the activation of diverse intracellular signaling pathways, including the nuclear factor kappa-light-chain-enhancer of activated B cells (NF-κB), mitogen-activated protein kinase (MAPK), and Jak-phosphoinositide 3-kinase (PI3K) pathways [1]. These pathways can trigger multiple biological events, such as proinflammatory cytokine and reactive oxygen species (ROS) production, intracellular trafficking, phagocytosis, and autophagy [7,8,9].

Recognition of *S*. Typhimurium is mediated mainly by TLR2, TLR4, and TLR5; however, TLR3 and TLR9 are also involved in their recognition [10]. TLR3 recognizes endosomal nucleic acids, such as viral and bacterial dsRNA, and the synthetic RNA analogs polyinosinic:polycytidylic acid (poly(I:C)) and polyinosine (poly(I)) [11]. Recognition of dsRNA through TLR3 leads to the recruitment of the adaptor protein TIR-domain-containing adapter-inducing interferon-β (TRIF), resulting in type I interferon (IFN) production [12]. Recent studies have shown that the TLR3-mediated pathway plays important roles in the control of *S*. Typhimurium infection, phagocytosis, and autophagy. Wong et al. showed that pretreatment with poly(I:C) promotes phagocytic uptake of *S*. Typhimurium and suppresses intracellular bacterial growth in the murine macrophage cell line RAW264.7, suggesting that TLR3 agonists lead to increased phagocytic capacity in murine macrophages [13]. Robinson also demonstrated that autophagy is not induced in *ifnar1*^−/−^ macrophages during *S*. Typhimurium infection, indicating that type I IFN positively regulates autophagy [14].

Eukaryotic cells maintain intracellular homeostasis through two major mechanisms [15]: one involves degradation of primarily short-lived proteins via the proteasome, while the other involves degradation of long-lived proteins or dysfunctional organelles via autophagy [16]. Autophagy is induced by changes in intracellular or extracellular environments, such as depletion of nutrients or invasion by pathogens [17]. At low levels, autophagy is understood to be a mechanism necessary for the maintenance of intracellular homeostasis and cell survival; however, excessive stimulation of autophagy leads to type II programmed cell death (PCD) [18]. During autophagy, unique double-membrane vesicles—called autophagosomes—form; these are hallmarks of autophagy. Autophagosomes completely surround dysfunctional organelles or pathogens in the cytoplasm and fuse with lysosomes to form autolysosomes. After the fusion of autophagosomes and lysosomes, targets within autophagosomes are degraded by lysosomal hydrolases from the autolysosomes [19]. Birmingham et al. showed that intracellular growth of *S*. Typhimurium is greater in murine embryonic fibroblasts (MEF) lacking the autophagy-related gene *Atg5* than in wild-type (WT) MEFs, indicating that autophagy contributes to the removal of invasive *S*. Typhimurium during infection [20]. Our previous study demonstrated that the TLR7 agonist imiquimod (IMQ) controls intracellular growth via autophagy in murine macrophages during *Mycobacterium tuberculosis* (Mtb) infection [21]. Although the TLR3-mediated pathway is known to induce autophagy [22,23], the molecular mechanism of this process with regard to the control of intracellular bacterial growth remains poorly understood. In this study, we found that stimulation of TLR3 with poly(I:C) significantly induced the conversion of LC3-1 to LC3-II in *S*. Typhimurium-infected murine macrophages. In addition, poly(I:C) suppressed the intracellular growth of *S*. Typhimurium via MEK-induced ROS production in murine macrophages. Poly(I:C) also increased nitric oxide (NO) production via activation of the NF-κB pathway, thus controlling intracellular *S*. Typhimurium growth during infection. These results indicate that poly(I:C) modulates intracellular bacterial growth by promoting autophagy in *S*. Typhimurium-infected cells, suggesting that poly(I:C)-induced autophagy may be a potential therapeutic target during *S*. Typhimurium infection.

## 2. Results

### 2.1. Treatment with Poly(I:C) Suppresses Intracellular Bacterial Growth in S. Typhimurium-Infected Murine Macrophages

**Figure 1:**
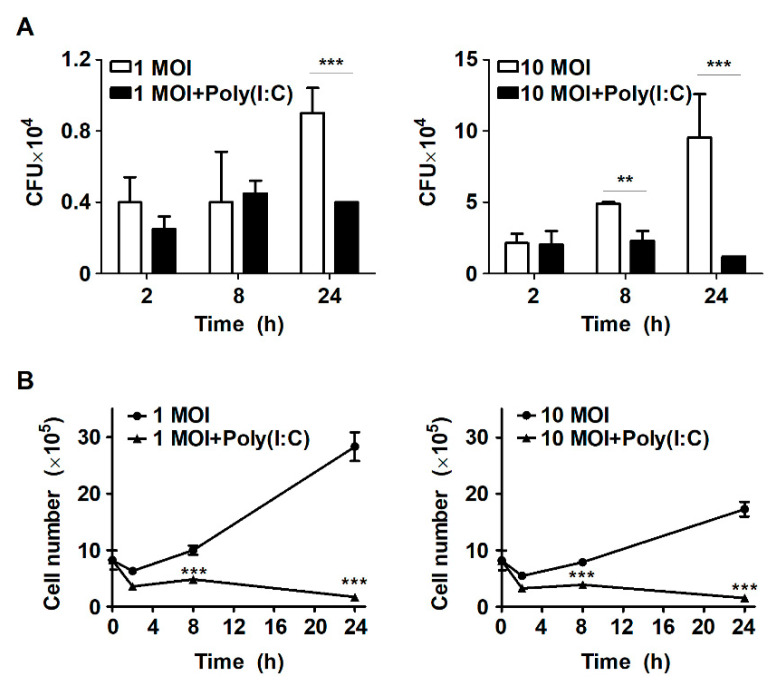
Poly(I:C) controls intracellular bacterial growth in *S*. Typhimurium-infected cells. (**A**) RAW264.7 cells were infected with *S*. Typhimurium at an MOI of 1 or 10 and then treated with poly(I:C) (10 μg/mL) for the indicated durations. Intracellular growth of *S*. Typhimurium was assessed by CFU assay. (**B**) The number of RAW264.7 cells was determined using a trypan blue exclusion assay. The data are presented as the mean ± SD (*n* = 3), and were analyzed via two-way ANOVA followed by Bonferroni’s post hoc test; ** *p* < 0.01, *** *p* < 0.001.

Our previous study demonstrated that stimulation with the TLR7 agonist IMQ enhances mycobactericidal activities via autophagy in murine macrophages during Mtb infection [21]. Accordingly, to determine whether stimulation by TLR agonists affects intracellular bacterial growth, RAW264.7 cells were stimulated with the TLR7 agonist IMQ or the TLR3 agonist poly(I:C) during *S*. Typhimurium infection. Intracellular bacterial growth was increased in a time-dependent manner in *S*. Typhimurium-infected macrophages (Figure 1A). However, intracellular bacterial growth was reduced by approximately twofold at 24 h after poly(I:C) treatment in cells infected with S. Typhimurium at an MOI of 1; however, it was similar to that of untreated cells at 2 h and 8 h (Figure 1A). These results were similar when cells were infected with *S*. Typhimurium at an MOI of 1 or 10 (Figure 1A). In cells infected with S. Typhimurium at an MOI of 10, treatment with poly(I:C) suppressed intracellular bacterial growth from 8 h, and showed a significant difference at 24 h (Figure 1A). In particular, poly(I:C)-treated cells exhibited approximately twofold and eightfold reductions in intracellular bacterial growth at 8 h and 24 h, respectively, compared to untreated cells (Figure 1A). In contrast, treatment with IMQ did not affect intracellular bacterial growth in *S*. Typhimurium-infected cells (data not shown). Next, we investigated the effect of poly(I:C) on host cell survival in *S*. Typhimurium-infected cells. Infection with *S*. Typhimurium did not affect cell survival until 24 h; however, host cell growth was significantly inhibited among poly(I:C)-treated cells compared to untreated cells (Figure 1B). Similarly, the cell growth rate was also reduced by more than threefold in IMQ-treated cells during *S*. Typhimurium infection (data not shown). These results suggest that poly(I:C) effectively controls the intracellular growth of *S*. Typhimurium in murine macrophages.

### 2.2. Poly(I:C) Activates the MAPK and NF-κB Signaling Pathways during S. Typhimurium Infection

**Figure 2:**
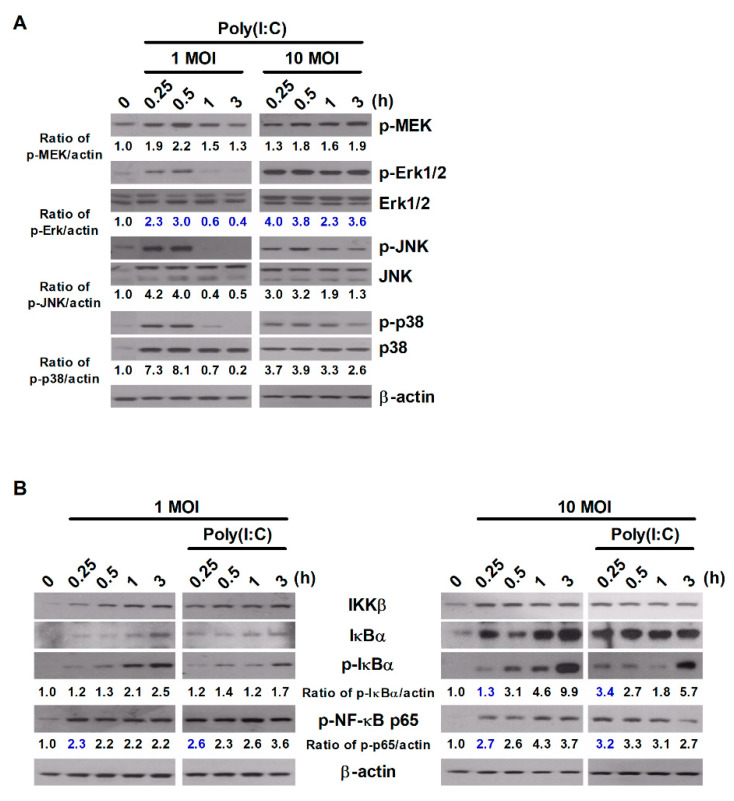
Poly(I:C) enhances the activation of MAPK and NF-κB signaling in *S*. Typhimurium-infected cells. RAW264.7 cells were infected with *S*. Typhimurium at an MOI of 1 or 10 and then treated with poly(I:C) (10 μg/mL) for the indicated durations. Total cell lysates were assessed via Western blot analysis to detect the expression of (**A**) MAPK-related proteins and (**B**) NF-κB-related proteins. Band intensities for phosphorylated proteins were quantified using ImageJ software and normalized to β-actin. Fold changes are indicated at the bottom of each lane.

*S*. Typhimurium expresses various ligands for PRRs, which activate diverse intracellular signaling cascades, including the NF-κB and MAPK signaling cascades [1]. Next, we investigated whether treatment with poly(I:C) triggers alterations in the intracellular signaling pathways in murine macrophages during *S*. Typhimurium infection. Poly(I:C) enhanced the phosphorylation levels of proteins involved in the MAPK and NF-κB signaling pathways in a manner dependent on the infectious dose of *S*. Typhimurium (Figure 2A,B). Treatment with poly(I:C) increased the phosphorylated levels of MAPK-related molecules—including MEK, Erk1/2, JNK, and p38—from 15 min in cells infected with *S*. Typhimurium at an MOI of 1 (Figure 2A). Unlike cells infected with *S*. Typhimurium at an MOI of 1, the phosphorylated levels of MAPK-related molecules were maintained for up to 3 h in cells infected with *S*. Typhimurium at an MOI of 10 after poly(I:C) treatment (Figure 2A). We found that phosphorylated level of Erk1/2 was most strongly enhanced in poly(I:C)-treated cells during *S*. Typhimurium infection, although the phosphorylation of p38 and JNK appeared to slightly increased (Figure 2A).

For molecules involved in the NF-κB signaling pathway, the phosphorylated levels of IκBα and NF-κB p65 were enhanced in an infectious dose- and time-dependent manner in cells infected with *S*. Typhimurium alone (Figure 2B). Treatment with poly(I:C) rapidly improved the phosphorylation of NF-κB p65 in cells with *S*. Typhimurium at an MOI of 10 compared to untreated cells at 15 min (Figure 2B). In particular, treatment with poly(I:C) increased the expression of phospho-MEK, phospho-Erk1/2, and phospho-NF-κB p65 at an early timepoint after treatment in *S*. Typhimurium-infected RAW264.7 cells compared to untreated cells (Figure 2A,B). These results indicate that poly(I:C) rapidly and strongly activates the MEK/Erk1/2 and NF-κB signaling pathways in murine macrophages during *S*. Typhimurium infection.

### 2.3. Poly(I:C) Induces Autophagy in S. Typhimurium-Infected Cells

**Figure 3:**
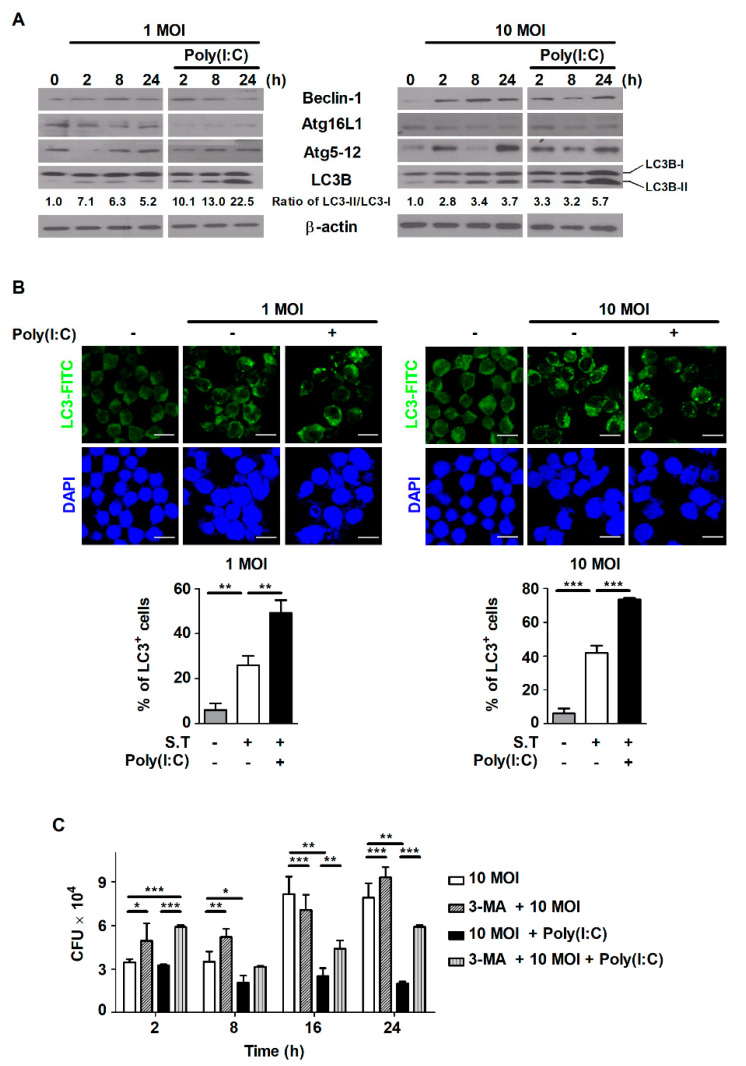
Poly(I:C) controls intracellular bacterial growth by inducing autophagy in *S*. Typhimurium-infected cells. RAW264.7 cells were infected with *S*. Typhimurium at an MOI of 1 or 10 and then treated with poly(I:C) (10 μg/mL) for the indicated durations. (**A**) Total cell lysates were assessed via Western blot analysis to detect the expression of autophagy-related proteins. The band intensity for LC3B-II was quantified using ImageJ software and normalized to LC3B-I. Fold changes are indicated at the bottom of each lane. (**B**) After poly(I:C) treatment, cells were stained with an anti-LC3 antibody, and intracellular LC3 (green) levels were monitored. The nuclei were stained with DAPI (blue). Upper panel: representative immunofluorescence images; scale bar = 20 μm. Bottom panel: bar graph showing the percentages of cells with LC3-positive puncta. (**C**) RAW264.7 cells were pretreated with 3-MA for 2 h and then infected with *S*. Typhimurium in the presence or absence of poly(I:C). Intracellular growth of *S*. Typhimurium was assessed via CFU assay. The data are presented as the mean ± SD (*n* = 3), and were analyzed via one-way ANOVA followed by Tukey’s post hoc test for multiple comparisons; * *p* < 0.05, ** *p* < 0.01, and *** *p* < 0.001.

Infection with *S*. Typhimurium did not affect host cell growth, whereas treatment with IMQ or poly(I:C) decreased the survival of *S*. Typhimurium-infected cells (Figure 1). We tried to identify whether the inhibition of cell growth resulting from poly(I:C) treatment was caused by autophagy—one of the mechanisms of PCD. We observed that the expression of autophagy-related proteins was enhanced by *S*. Typhimurium infection, depending on the infection level and infection time, in RAW264.7 cells (Figure 3A). In particular, the conversion of LC3-I to LC3-II—a hallmark of autophagy that is correlated with autophagy levels—was significantly increased in poly(I:C)-treated cells compared to untreated cells during *S*. Typhimurium infection (Figure 3A). To further monitor whether poly(I:C) enhances autophagy during *S*. Typhimurium infection, we performed LC3 immunofluorescence analysis. As shown in Figure 3B, infection with *S*. Typhimurium markedly enhanced the percentage of endogenous LC3-positive cells in an infectious-dose-dependent manner. Compared to no treatment, treatment with poly(I:C) also significantly increased the percentage of cells with LC3-positive puncta during *S*. Typhimurium infection (Figure 3B). To demonstrate whether poly(I:C) controls intracellular bacterial growth by inducing autophagy in *S*. Typhimurium-infected cells, cells were pretreated with the autophagy inhibitor 3-MA. Intracellular bacterial growth, which was reduced by poly(I:C), was restored in 3-MA-pretreated cells during *S*. Typhimurium infection (Figure 3C). These data demonstrate that poly(I:C) contributes to the control of intracellular bacterial growth via autophagy in *S*. Typhimurium-infected cells.

### 2.4. Poly(I:C) Enhances the Production of ROS and NO in S. Typhimurium-Infected Cells

**Figure 4:**
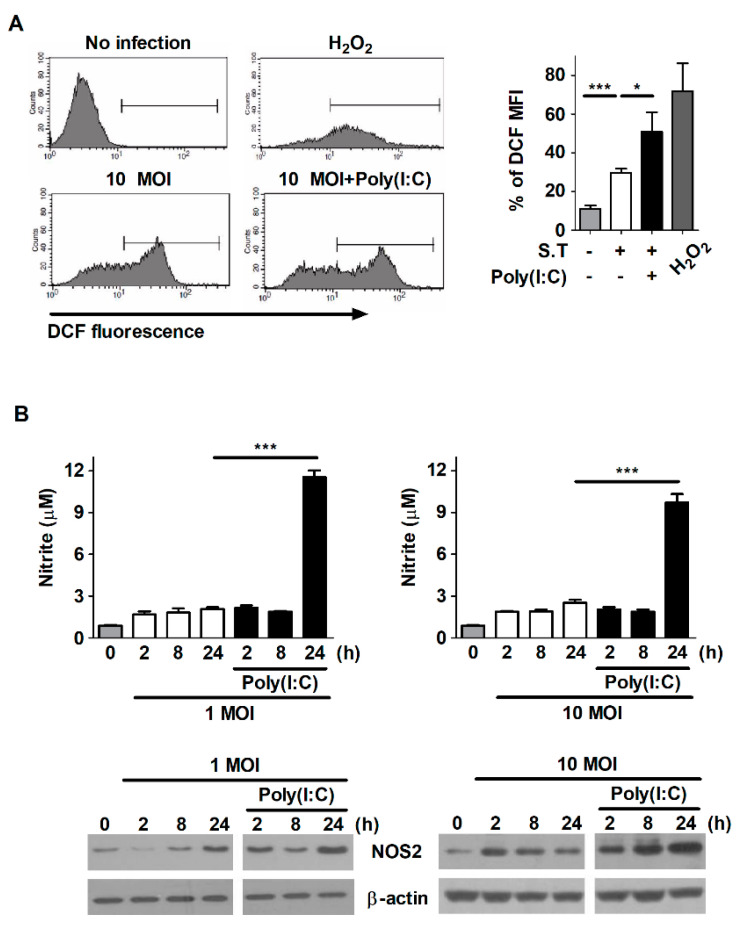
Poly(I:C) increases the production of ROS and NO in *S*. typhimurium-infected cells. (**A**) RAW264.7 cells were infected with *S*. Typhimurium at an MOI of 1 or 10 and then treated with poly(I:C) (10 μg/mL) for 30 min. The cells were labeled with 5-[and-6]-chloromethyl-2′,7′-dichlorodihydrofluorescein diacetate (DCFH-DA) and then measured via flow cytometry. The bar graph indicates the percentage of DCF mean fluorescence intensity (MFI). H_2_O_2_ was used as a positive control. (**B**) RAW264.7 cells were infected with *S*. Typhimurium at an MOI of 10 and then treated with poly(I:C). NO production was assessed in cell culture supernatants at the indicated timepoints. Total cell lysates were assessed via Western blot analysis to detect the expression of iNOS. The data are presented as the mean ± SD (*n* = 3), and were analyzed via one-way ANOVA followed by Tukey’s post hoc test for multiple comparisons; * *p* < 0.05 and *** *p* < 0.001.

The generation of ROS and NO plays a role in the restriction of intracellular pathogens, including *S*. Typhimurium, Mtb, and *Listeria monocytogenes* [24,25,26]. Therefore, we investigated whether poly(I:C) increases the production of ROS and NO in macrophages during *S*. Typhimurium infection. Infection with *S*. Typhimurium increased ROS production in RAW264.7 cells; however, ROS production was significantly greater in poly(I:C)-treated cells than in untreated cells during *S*. Typhimurium infection (Figure 4A). The secretion of NO was also markedly greater in poly(I:C)-treated cells than in untreated cells at 24 h after *S*. Typhimurium infection (Figure 4B). This difference was consistent with the expression levels of iNOS, which is a key enzyme generating NO (Figure 4B). Our data indicate that poly(I:C) enhances the production of ROS and NO in *S*. Typhimurium-infected macrophages.

### 2.5. Poly(I:C) Controls Intracellular Bacterial Growth by Increasing the Production of ROS through the MEK1/2-Mediated Pathway in S. Typhimurium-Infected Macrophages

**Figure 5:**
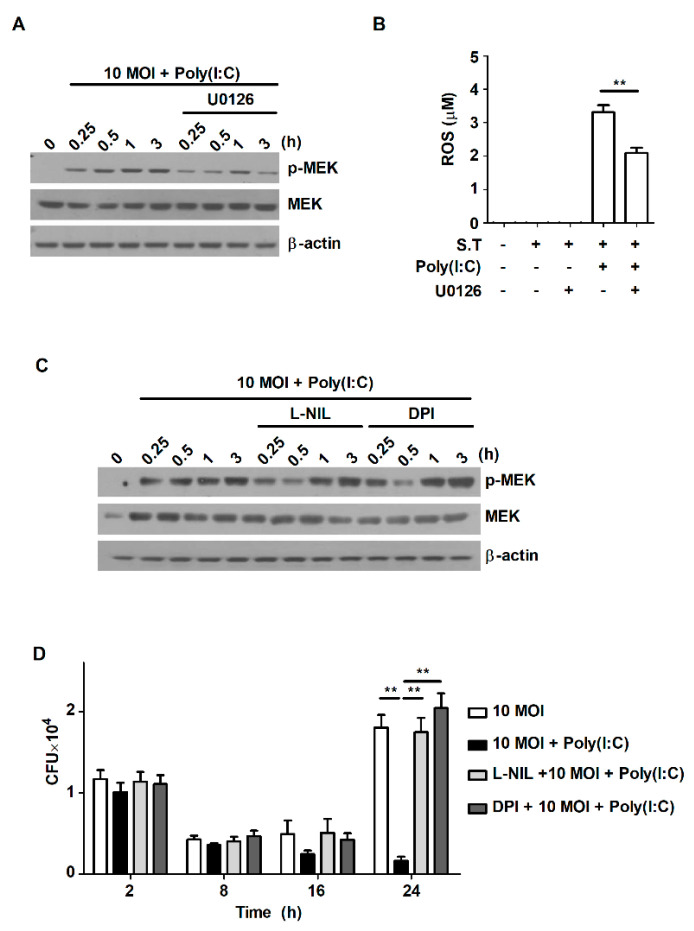
Poly(I:C) suppresses intracellular bacterial growth by promoting ROS production via MEK1/2-mediated signaling in *S*. Typhimurium-infected cells. (**A**,**B**) RAW264.7 cells were pretreated with U0126 for 1 h and then infected with *S*. Typhimurium in the presence or absence of poly(I:C). (**A**) Total cell lysates were assessed via Western blot analysis to detect phospho-MEK1/2 at the indicated timepoints. (**B**) ROS production was determined in cell culture supernatants. (**C**,**D**) RAW264.7 cells were pretreated with DPI or L-NIL for 1 h and then infected with *S*. Typhimurium in the presence or absence of poly(I:C). (**C**) Total cell lysates were assessed via Western blot analysis to detect phospho-MEK1/2 at the indicated timepoints. (**D**) Intracellular growth of *S*. Typhimurium was assessed via CFU assay. The data are presented as the mean ± SD (*n* = 3), and were analyzed with one-way ANOVA followed by Tukey’s *post* hoc test for multiple comparisons; ** *p* < 0.01.

Our results showed that treatment with poly(I:C) triggered activation of the MAPK signaling pathway and ROS production during *S*. Typhimurium infection (Figure 2A and Figure 4A). These results are consistent with a number of previously published studies showing that poly(I:C) induces ROS production [27,28,29]. Stimulation by TLR agonists themselves can induce ROS production; however, ROS are also generated through TLR-induced intracellular signaling pathways. It has been reported that infection with *S*. Typhimurium activates MAPK to generate ROS production [30]. Therefore, we attempted to investigate the correlation between ROS production and the MEK1/2-mediated signaling pathway, which is activated from an early stage after *S*. Typhimurium infection. To identify whether treatment with poly(I:C) affects ROS production via the MEK1/2-mediated signaling pathway in *S*. Typhimurium-infected cells, cells were pretreated with the MEK/Erk inhibitor U0126. Treatment with poly(I:C) significantly increased the phosphorylation level of MEK1/2 at the early timepoints after *S*. Typhimurium infection, but the increase was attenuated by the inhibition of MEK1/2 using U0126 (Figure 5A). U0126 treatment significantly blocked ROS production in poly(I:C)-treated cells during *S*. Typhimurium infection (Figure 5B). However, inhibition of ROS production with DPI—an ROS scavenger—did not affect the phosphorylation level of MEK1/2 in poly(I:C)-treated cells during *S*. Typhimurium infection (Figure 5C). Pretreatment with the selective iNOS inhibitor L-NIL also did not affect the expression of phospho-MEK1/2 in poly(I:C)-treated cells during *S*. Typhimurium infection (Figure 5D). These results indicate that poly(I:C) triggers ROS production via the MEK1/2-mediated signaling pathway in *S*. Typhimurium-infected cells.

## 3. Discussion

*Salmonella enterica* (*S. enterica*) is a pathogen that causes serious diseases worldwide, including food poisoning, intestinal fever, sepsis, and gastroenteritis. *S. enterica* is classified into more than 2000 serovars in accordance with its surface antigen structures, host range, and ability to induce disease [31]. *S*. Typhimurium isolated from rodents is a flagellated, Gram-negative pathogen that causes systemic infection in mice, resembling typhoid fever caused by *S. enterica* in humans [1]. Thus, murine models of *S*. Typhimurium have been used to study human typhoid infections. *S*. Typhimurium expresses various PAMPs, including flagellin, LPS, and CpG DNA [10]. PAMPs from *S*. Typhimurium can be recognized by PRRs such as TLRs, NLRs, and RLRs. It is well known that *S*. Typhimurium with flagellin is recognized by TLR5 [32]. TLRs are the key sensors for PAMPs, and play important roles in host defense against pathogens [33]. For example, TLRs contribute to the control of intracellular bacterial survival via upregulation of phagocytosis, antigen presentation, and cytokine production [34,35]. Published reports have shown that several TLR agonists enhance the phagocytic ability of macrophages during *S*. Typhimurium infection [13,36,37,38,39]. In line with these reports, our previous study demonstrated that treatment with the TLR7 agonist IMQ induces autophagy—a cell death mechanism—and controls intracellular Mtb growth in murine macrophages [21]. IMQ is an immunotherapeutic agent with immune-stimulating capabilities. According to previous studies, IMQ promotes antiviral innate immunity and T_H_1-mediated adaptive immune responses [40]. In addition, our previous study demonstrated that IMQ limits the development of melanoma through autophagic cell death during radiotherapy for melanoma [41]. In this context, it has been reported that IMQ induces autophagic cell death in the human colon carcinoma cell line Caco-2, as well as a basal cell carcinoma cell line (BCC/KMC1) [42,43]. Therefore, IMQ can function as an immunomodulatory agent with both anticancer and antibacterial activities. However, each TLR can exhibit different functions in pathogen elimination; therefore, the contributions of individual TLRs to host defense against *S*. Typhimurium remain poorly understood. TLR3 is expressed in endosomal compartments, similarly to TLR7. Poly(I:C) is an agonist that can stimulate TLR3, and has antiviral and anticancer properties [44]. Lantier et al. found that administration of poly(I:C) reduced intestinal infection caused by the parasite *Cryptosporidium parvum* in neonatal mice [45], suggesting that poly(I:C) protects neonatal mice from parasitic infection. However, it is not well known whether stimulation of TLR3 with poly(I:C) contributes to the control of bacterial pathogens, although TLR3 is expressed in the endosomal compartments along with TLR7. In this study, we aimed to determine the contributions of TLR agonists to antibacterial activity against *S*. Typhimurium infection. Infection with *S*. Typhimurium increased bacterial and cell growth in a time-dependent manner; however, treatment with poly(I:C)—a TLR3 agonist—decreased cell growth and intracellular bacterial growth by approximately 3–5-fold. Our data suggest that poly(I:C) can control intracellular bacterial growth in *S*. Typhimurium-infected murine macrophages.

Exposure to *S*. Typhimurium can also induce intestinal inflammation in mice via various intracellular signaling pathways [6]. Upon infection with *S*. Typhimurium, recognition by TLRs activates various intracellular signaling pathways—such as the NF-κB and MAPK pathways—via downstream signaling molecules [1]. The activation of intracellular signaling pathways contributes to the elimination of invading pathogens by inducing recruitment of innate immune cells through increased production of ROS, NO, and multiple cytokines. For example, a previous study showed that infection with *S*. Typhimurium activates the MAPK signaling pathway to generate ROS and NO [30]. Consistent with this finding, treatment with poly(I:C) enhanced the activation of MEK1/2 and Erk1/2 at early timepoints in *S*. Typhimurium-infected RAW264.7 cells. In addition, the production of ROS and NO was significantly increased in poly(I:C)-treated cells during *S*. Typhimurium infection. We tried to determine which events preferentially occur between the activation of the MEK1/2-mediated signaling pathway and the production of ROS and NO in poly(I:C)-treated cells during *S*. Typhimurium infection. Blockade of MEK/Erk1/2 with U0126 suppressed ROS production. However, inhibition of ROS production did not alter the activation of MEK1/2, and increased intracellular bacterial growth in poly(I:C)-treated cells during *S*. Typhimurium infection. Our results indicate that poly(I:C) controls intracellular bacterial growth by enhancing ROS production through the activation of MEK1/2 during *S*. Typhimurium infection.

Infection with *S*. Typhimurium can trigger autophagy [46]. Autophagy is a homeostatic mechanism that removes invading pathogens through degradation in autophagosomes [47]. Birmingham et al. demonstrated that colocalization between LC3-positive bacteria and LAMP-1 decreases in Atg5-deficient MEFs during *S*. Typhimurium infection [20]; in addition, they found that Atg5-deficient MEFs display uncontrolled intracellular growth of *S*. Typhimurium, indicating that autophagy plays an important role in controlling intracellular *S*. Typhimurium growth during infection. Xu et al. first revealed that stimulation of TLR4 with LPS induces autophagy, and it has been reported that not only TLR4, but also TLR7, can activate autophagy [21,47,48,49]. These studies suggest that TLR agonists can regulate autophagy, which functions as a defense mechanism against bacterial infection. Here, we also found that the TLR3 agonist poly(I:C) may be a candidate molecule for the enhancement of defense mechanisms that control intracellular bacterial growth via the induction of autophagy during *S*. Typhimurium infection. These results support the idea that TLRs are closely related to the regulation of autophagy.

Therefore, TLR3 agonist treatment has two key effects on the innate immune response against *S*. Typhimurium infection: First, TLR3 agonist treatment enhances ROS production via an MEK1/2-mediated signaling pathway in *S*. Typhimurium-infected macrophages. Second, TLR3 agonist treatment activates autophagy during *S*. Typhimurium infection. These processes function together to restrict intracellular bacterial growth via the innate immune response against *S*. Typhimurium infection.

## 4. Materials and Methods

### 4.1. Reagents

The TLR3 agonist poly(I:C) was purchased from Sigma-Aldrich (Burlington, MA, USA). The autophagy inhibitor 3-methyladenine (3-MA) was also purchased from Sigma-Aldrich. The MEK1/2 inhibitor U0126 was purchased from Cell Signaling Technology (Danvers, MA, USA). The NADPH oxidase inhibitor diphenyleneiodonium (DPI) and the inducible nitric oxide synthase (iNOS) inhibitor L-N6-(1-iminoethyl)-lysine (L-NIL) were both purchased from EMD Chemicals, Inc. (Gibbstown, NJ, USA).

### 4.2. Cell Culture

The murine macrophage cell line RAW264.7 was purchased from the American Type Culture Collection (ATCC, Manassas, VA, USA), and was maintained in RPMI 1640 culture medium (Corning Cellgro, New York, NY, USA) containing 10% fetal bovine serum (FBS; Lonza, Walkersville, MD, USA) and penicillin/streptomycin (Gibco-BRL, Gaithersburg, MD, USA) at 37 °C with 5% CO_2_.

### 4.3. S. Typhimurium Culture Conditions

The *S*. Typhimurium strain ATCC14028 was used for all experiments in this study. *S*. Typhimurium was inoculated on LB agar9 (tryptone (BD Biosciences, Franklin Lakes, NJ, USA), yeast extract powder (USB, Cleveland, OH, USA), sodium chloride (Bio Basic Inc., New York, NY, USA), and agar powder (DaeJung Chemicals & Metals Co., Ltd., Gyeonggi-do, Korea)) and incubated at 37 °C overnight. A single colony of *S*. Typhimurium was inoculated and cultured in 5 ml of LB broth with vigorous shaking until an optical density at 600 nm (OD_600_) of 1 (~1 × 10^9^ cfu/mL) was reached.

### 4.4. S. Typhimurium Infection In Vitro

RAW264.7 cells were seeded at a density of 1 × 10^4^ cells/well in 6-well cell culture plates (SPL Lifesciences, Gyeonggi-do, Korea) and incubated for 2 days at 37 °C with 5% CO_2_. The cells were washed twice with phosphate-buffered saline (PBS; Gibco Life Technologies, MA, USA) and infected with *S*. Typhimurium at a multiplicity of infection (MOI) of 1 or 10. The cell culture plates were centrifuged at 500× *g* for 5 min and incubated for 30 min at 37 °C with 5% CO_2_. Then, the cells were washed twice with PBS to remove extracellular bacteria and treated with the TLR3 agonist poly(I:C) (10 μg/mL) in the presence of gentamicin (60 μg/mL).

### 4.5. Cell Treatments

For the inhibition of autophagy, RAW264.7 cells were pretreated with 3-MA (10 mM) for 2 h. To inhibit the production of ROS or NO, cells were pretreated with DPI (10 μM) or L-NIL (30 μM) for 1 h. For the inhibition of the MEK1/2-mediated signaling pathway, cells were pretreated with U0126 (10 μM) for 1 h. RAW264.7 cells were pretreated with each inhibitor for the indicated time periods and then washed twice with PBS. Cells were infected with *S*. Typhimurium at an MOI of 1 or 10 for 30 min and then washed twice with PBS. Then, the cells were treated with poly(I:C) (10 μM) for the indicated time periods.

### 4.6. Cell Viability Assay

RAW264.7 cells (1 × 10^4^ cells/well) were infected with *S*. Typhimurium at an MOI of 1 or 10 for 30 min and then washed with PBS twice. Then, the cells were treated with poly(I:C) (10 μg/mL) in the presence of gentamicin. The number of viable cells was assessed using a trypan blue exclusion assay.

### 4.7. Colony-Forming Unit (CFU) Assay

A CFU assay was performed as previously described [50]. Briefly, 1 × 10^4^ RAW264.7 cells were infected with *S*. Typhimurium for 30 min and then washed with PBS twice to remove extracellular bacteria. Then, the cells were treated with poly(I:C) (10 μg/mL) in the presence of gentamicin for the indicated durations. At the indicated timepoints, cells were washed twice with PBS and then treated with 200 μL of PBS containing 1% Triton X-100 to isolate intracellular bacteria for 10 min at 37 °C with 5% CO_2_. To determine the number of intracellular bacteria, cell lysates were serially diluted and inoculated into LB agar. Then, the LB agar plates were incubated at 37 °C for 20 h, and the colonies were counted.

### 4.8. Western Blot Analysis

RAW264.7 cells were infected with *S*. Typhimurium at an MOI of 1 or 10 and treated with poly(I:C) for the indicated durations. Cells were collected and lysed with RIPA buffer in the presence of a protease inhibitor cocktail. The composition of the RIPA buffer and the method of protein isolation have been described previously [51]. The concentrations of the extracted proteins were measured via Bradford assay. The extracted proteins were loaded onto an appropriate SDS-PAGE gel (10–15%), depending on the protein size. The separated proteins in the gel were transferred to a PVDF membrane at 120 V for 1 h, and then Western blot analysis was performed according to a method described in detail in our previous study. Anti-LC3B, anti-Atg12-5 complex, anti-Atg16L1, anti-Beclin-1, anti-phospho-MEK1/2, anti-MEK1/2, anti-phospho-ERK1/2, anti-ERK1/2, anti-phospho-JNK1/2, anti-JNK1/2, anti-phospho-p38, and anti-p38 antibodies were purchased from Cell Signaling Technology. Anti-NOS2, anti-IKKβ, anti-phospho-IκB-α, anti-IκB-α, anti-phospho-NF-κB p65, and anti-actin antibodies were purchased from Santa Cruz Biotechnology, Inc. (Delaware Avenue, Philadelphia, PA, USA). An anti-actin antibody was used as a loading control. All blots were detected in cropped membranes according to protein size. All blots in membranes were treated with enhanced chemiluminescence (ECL) reagent and exposed to Kodak XAR film in a darkroom. Band intensities were quantified using ImageJ software and normalized to β-actin. Fold changes in band intensities are indicated at the bottom of each lane.

### 4.9. Immunofluorescence Analysis

Immunofluorescence analysis was performed as previously described [21]. Briefly, 5 × 10^4^ RAW264.7 cells were seeded on coverslips in 12-well cell culture plates and incubated for 2 days at 37 °C with 5% CO_2_. After incubation, the cells were infected with *S*. Typhimurium and then treated with poly(I:C). At the indicated timepoints, the cells were washed with PBS and fixed in PBS containing 4% paraformaldehyde for 15 min at room temperature. To permeabilize the cell membrane, the cells were lysed with PBS containing 0.2% Triton X-100 for 15 min at room temperature. The cells were then stained with an anti-LC3 antibody (MBL, Nagoya, Japan) for 2 h, followed by an FITC-conjugated secondary antibody. The nuclei were stained with 4′-6-diamidino-2-phenylindole (DAPI, Sigma-Aldrich, St. Louis, MO, USA) for 5 min and washed with PBS 4 times. The coverslips were mounted onto slides with a drop of Fluoromount-G^TM^ mounting medium (Southern Biotech, Birmingham, AL, USA) and observed via confocal microscopy (FV1000 SPD; Olympus, Japan). To quantify autophagic flux, a total of 3–5 fields were randomly selected, and the number of LC3-positive cells was normalized to the total number of cells. The data are representative of three independent experiments conducted in triplicate.

### 4.10. Flow Cytometry

Cell-permeant 2′,7′-dichlorodihydrofluorescein diacetate (DCF-DA) was used to measure intracellular ROS production. RAW264.7 cells were infected with *S*. Typhimurium and treated with poly(I:C). Then, the cells were loaded with 10 μM DCF-DA for 20 min at 37 °C with 5% CO_2_ and washed with cold PBS 3 times. The DCF fluorescence signal was detected using a FACSCalibur flow cytometer (BD Biosciences, NJ, USA). All FACS data were analyzed using CellQuest software.

### 4.11. ROS Detection Assay

ROS production was measured using a QuantiChrom peroxide assay kit (BioAssay Systems, Hayward, USA) according to the manufacturer’s instructions. The concentrations of ROS in cell culture supernatants were quantified by measuring the absorbance at 585 nm using a microplate reader (BioTek Instruments Inc., Winooski, VT, USA).

### 4.12. NO Detection Assay

NO was measured using an NO detection kit according to the manufacturer’s instructions (iNtRON Biotechnology, Gyeonggi-do, Korea), and as described in our previous study [52]. In brief, RAW264.7 cells were infected with *S*. Typhimurium and treated with poly(I:C) for the indicated times. The cell culture supernatant was collected and centrifuged at 6000 rpm for 10 min. Then, the cells were incubated sequentially with sulfanilamide in reaction buffer (N1 buffer) and naphthylethylenediamine in stabilizer buffer (N2 buffer) for 10 min. The concentrations of nitrite in the cell culture supernatants were detected at 540 nm using a microplate reader (BioTek Instruments Inc., Winooski, VT, USA). A standard curve was created with purified nitrite.

### 4.13. Statistical Analysis

All data were obtained from three independent experiments conducted in triplicate. Statistical significance was determined by Student’s *t*-test or one-way ANOVA followed by Tukey’s post hoc test, or by two-way ANOVA followed by Bonferroni’s post hoc test, using GraphPad Prism 5 (GraphPad Software Inc., San Diego, CA, USA). Differences were considered significant at * *p* < 0.05; ** *p* < 0.01, and *** *p* < 0.001; ns is used to indicate that a difference was not significant (*p* > 0.05).

## Data Availability

The data presented in this study are available in article. Raw data will be provided from the corresponding author on reasonable request.
